# Digital Transcriptome Analysis of Putative Sex-Determination Genes in Papaya (*Carica papaya*)

**DOI:** 10.1371/journal.pone.0040904

**Published:** 2012-07-16

**Authors:** Naoya Urasaki, Kazuhiko Tarora, Ayano Shudo, Hiroki Ueno, Moritoshi Tamaki, Norimichi Miyagi, Shinichi Adaniya, Hideo Matsumura

**Affiliations:** 1 Okinawa Prefectural Agricultural Research Center, Itoman, Japan; 2 Gene Research Center, Shinshu University, Ueda, Japan; 3 University of the Ryukyus, Nishihara, Japan; Centro de Investigación y de Estudios Avanzados del IPN, Mexico

## Abstract

Papaya (*Carica papaya*) is a trioecious plant species that has male, female and hermaphrodite flowers on different plants. The primitive sex chromosomes genetically determine the sex of the papaya. Although draft sequences of the papaya genome are already available, the genes for sex determination have not been identified, likely due to the complicated structure of its sex-chromosome sequences. To identify the candidate genes for sex determination, we conducted a transcriptome analysis of flower samples from male, female and hermaphrodite plants using high-throughput SuperSAGE for digital gene expression analysis. Among the short sequence tags obtained from the transcripts, 312 unique tags were specifically mapped to the primitive sex chromosome (X or Y^h^) sequences. An annotation analysis revealed that retroelements are the most abundant sequences observed in the genes corresponding to these tags. The majority of tags on the sex chromosomes were located on the X chromosome, and only 30 tags were commonly mapped to both the X and Y^h^ chromosome, implying a loss of many genes on the Y^h^ chromosome. Nevertheless, candidate Y^h^ chromosome-specific female determination genes, including a MADS-box gene, were identified. Information on these sex chromosome-specific expressed genes will help elucidating sex determination in the papaya.

## Introduction

Papaya (*Carica papaya*) is a trioecious species with male, female and hermaphrodite flowers on separate plants. It is primarily cultivated in tropical and subtropical areas. Generally, the pear-shaped fruits from hermaphrodite flowers are commercially preferred, although the female flowers can also produce fruits if pollinated. Currently, there are several critical problems in the breeding and cultivation of hermaphrodite plants. First, genetically fixing hermaphrodite characteristics is impossible due to the segregation of sex type, even when self-fertilized (see below). Thus, efforts to optimize hermaphrodite selection among the segregates are essential in the field. Second, under high summer temperatures, the flowers have been observed to change from hermaphrodite to male because of ovary abortion and abnormal carpelloid or pentandoria flowers [1], [2]. These problems are due to the complicated genetic control of sex in the papaya; therefore, identifying the genes and elucidating the mechanisms involved in sex determination is necessary to overcome the practical obstacles in papaya cultivation.

Using the results from an analysis of the segregation of sex types after cross- or self-pollination, a genetic model of papaya sex determination was hypothesized based on three alleles (*M, M^h^* and *m*) at a single locus, *Sex1* [3], [4]. At this locus, females carry only the recessive male-sterility allele *m*, and therefore their genotype is *mm*. Males are heterozygous for *m* and a dominant female-suppressing allele, *M*, and therefore their genotype is *Mm*. Hermaphrodites possess an independent allele, *M^h^*, and their genotype is *M^h^ m*. Individuals homozygous for the dominant alleles (*MM* and *M^h^ M^h^*) and the *M M^h^* heterozygote are lethal. However, one study has suggested that a complex of genes rather than a single gene determines the sex of papaya [5], and a cytological study of the pollen mother cell demonstrated the presence of sex chromosomes in the papaya [6]. Recent progress, including the high-density genetic mapping and sequencing of bacterial artificial chromosomes (BACs) of the papaya genome, has revealed the existence of a pair of primitive sex chromosomes and the significant suppression of recombination near the putative sex-determination locus [7], [8]. The primitive sex chromosome controlling male determination is designated as the Y chromosome, and it is paired with the X chromosome. The male-specific region on the Y chromosome (MSY) suppresses recombination between the X and Y chromosomes [9]. In this system, the Y^h^ chromosome controls hermaphrodite sex determination. Both the X and Y^h^ chromosomes contain an approximately 8–9 Mb MSY with low gene density and a highly repetitive sequence [10], [11], [12]. The DNA markers responsible for the genetic discrimination of females, males and hermaphrodites were mapped to the MSY [13]. Females are homogametic for the X chromosome (XX), whereas males and hermaphrodites are heterogametic, possessing the XY and X Y^h^ chromosomes, respectively.

Sex chromosomes have been identified in 48 plant species [14], and the evolution of these plant sex chromosomes has been predicted [15]. According to the model of sex-chromosome evolution, male- and female-sterile mutations with complementary dominance occur in close proximity on a chromosome. Recombination suppression between these two sterility loci within the MSY region facilitates the divergence of the X and Y sex chromosomes. By comparison, the genomic sequences in the MSY regions of the X and Y^h^ chromosomes were frequently rearranged, and the genes on the Y (Y^h^) chromosome were lost due to the accumulation of mutations. A comparison of the partial genomic sequences of the Y and Y^h^ chromosomes revealed high sequence similarity [11], and they are predicted to have diverged 73,000 years ago [16]. Genetic and molecular approaches have been used to elucidate the mechanisms of sex determination in some plants, including the *Silene* spp. [17], [18], [19]. Nevertheless, the genes for sex determination have not yet been identified.

In papaya, several genes were identified in the MSY regions on both the Y and X chromosomes, but they did not show differential expression among the sex types [11]. Defining the candidate genes for sex determination using fine genetic mapping or genomic sequences of the MSY region is difficult due to the increased frequency of retrotransposons and redundant sequences [12]. Thus, we have employed global gene expression analysis (transcriptome analysis) to identify active genes on the papaya sex chromosome. For this study, a high-throughput (Ht-) SuperSAGE analysis [20] was performed using flower buds from male, female and hermaphrodite papaya flowers to examine the transcripts from the sex chromosomes. Ht-SuperSAGE is a method of digital gene expression profiling that involves the isolation of 26-bp tag fragments from expressed transcripts [21], [22], [23]. Using a combination of genetic information (mapping of the MSY region), genomic sequences and transcriptome data, we identified candidate genes for sex determination in the papaya.

## Results

### SuperSAGE Analysis of Flower Buds from the Three Sex Types in Papaya

**Figure 1:**
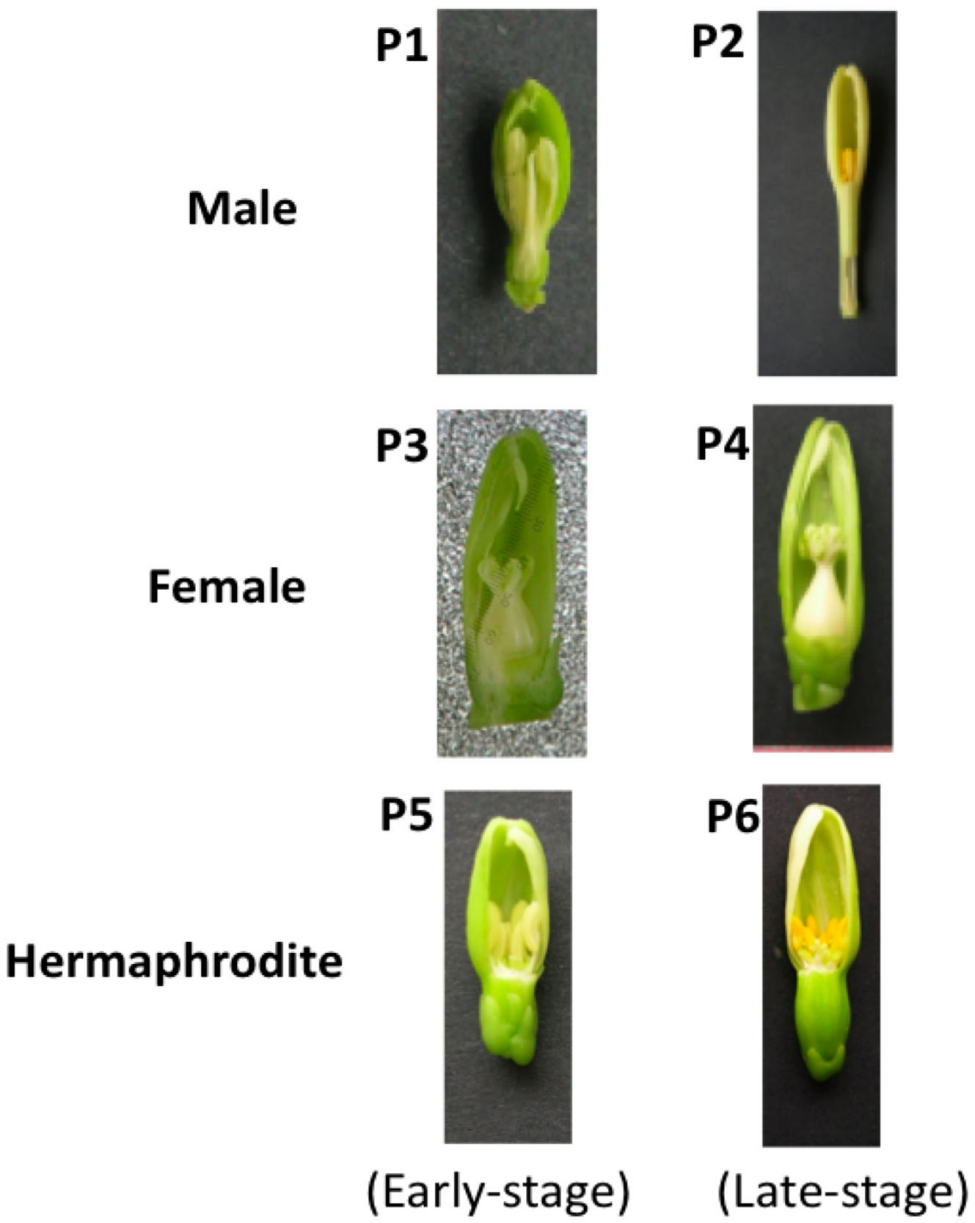
Flower samples for the Ht-SuperSAGE analysis. The papaya flowers used for the RNA extraction and Ht-SuperSAGE analysis (P1: male 7-mm flower; P2: male 20-mm flower; P3: female 7-mm flower; P4: female 20-mm flower; P5: hermaphrodite 7-mm flower; and P6: 20-mm hermaphrodite flower).

To identify candidate sex-determination genes in papaya, we conducted a large-scale transcriptome analysis of flowers from the three sex types using the Ht-SuperSAGE method. RNA was extracted from flowers at two developmental stages in the male (from TM1), female and hermaphrodite (from Sunrise Solo) plants as described in the Methods section, and these flower samples were designated as P1 to P6, respectively (Figure 1, Table 1). At the early stage of flower development (when the flower buds are no more than 7 mm in length), pistils that were morphologically similar to those in the hermaphrodite flowers were observed even in the male flowers (Figure 1). The primitive pistils in the male flowers were degenerated at later stages of development (flower buds 20 mm in length) when the male, female and hermaphrodite flowers were differentiated. Therefore it was expected that the genes for sex determination or differentiation could potentially be identified through a comparison of the transcriptome among these samples. Double-stranded cDNA was synthesized from the six papaya flower RNA samples and used for tag extraction from NlaIII sites (5′-CATG) in the cDNA using the EcoP15I restriction enzyme as described in the Methods section. The tag extraction and PCR amplification were performed according to the original protocol for the Ht-SuperSAGE analysis using the Illumina Genome Analyzer IIx [20], except that the adapter sequences for the SOLiD3 and the index sequences were used to distinguish the tag sequences from the raw sequence data of the pooled samples. The six-base index sequences used for the analyzed samples are listed in Table 1. The amplified tag fragments were pooled with the indexed adapters and subjected to SOLiD3 sequencing.

**Table 1:** Summary of the Ht-SuperSAGE analysis.

Sample code*	Flower buds analyzed	Indexsequence	Number oftotal tags	Number ofunique tags	Number ofnon-singleton tags
P1	Male early-stage	ACACAA	1,886,181	190,431	43,441
P2	Male late-stage	ATAGAG	1,585,205	200,775	42,921
P3	Female early-stage	AGAGTG	1,857,405	390,915	54,694
P4	Female late-stage	AGGCTG	1,410,279	179,994	40,297
P5	Hermaphrodite early-stage	CCGAAG	1,130,499	113,254	32,657
P6	Hermaphrodite late-stage	CGGATG	1,404,175	139,087	39,505
	(Total)		9,273,744	1,214,456	253,515

* Sample code was designated in Figure 1.

After converting the raw sequence data to the FASTA format, the reads containing the exact index sequences and the NlaIII site were selected. In total, 9,273,744 SuperSAGE tag-encoding sequences were selected and classified into six groups according to the index sequences that corresponded to each sample. Subsequently, the 26-bp tags were extracted from the NlaIII sites, and their counts were calculated to obtain an expression (tag) profile for each sample. The total tag counts and numbers of the unique tags in each sample are shown in Table 1. More than one million tags (with an average of 1,545,624 tags per sample) were obtained from each of the samples, which contained more than 30,000 non-singleton tags (unique tags with a count greater than two). Considering the predicted number of genes (22,934 genes) in the papaya genome [12], almost all of the expressed genes could be observed in this analysis.

### Expressed Genes on the Sex Chromosomes

By comparing the tag profiles among the six samples (P1–P6), many differentially expressed tags were identified (Table S1). These tags might correspond to genes involved in the development of the male or female floral organs or to genes crucial for sex differentiation or sex determination. To extract the genes responsible for sex determination, we focused on the tags that mapped to the MSY region on each sex chromosome. Tags occurring more than 10 times among the data from all of the samples were selected as queries in a BLASTN search against the BAC clone sequences of the MSY region. We selected sequences of the BAC clones for the MSY region from the Y^h^ and X chromosomes from GenBank (Table S2) and designated these sequences as sex-chromosome sequences in the present study. These sequences were obtained from the genome of a hermaphrodite plant of the cultivar SunUp, which was genetically similar to the plant material used in this study (cultivar Sunrise) was the source of these sequences [12]. In total, 456 unique tag sequences showed a perfect match to the BAC clone sequences of the sex chromosomes (X or Y^h^). These perfectly matched tags were designated as SC-tags (sex-chromosome tags, Table S3, S4, S5). Among these, 312 tags were uniquely mapped to the sex chromosomes (Table S3). The remaining SC-tags showed an additional perfect match to nuclear genomic sequences other than the sex chromosomes (133 tags in Table S4) or to chloroplast or mitochondrial genomic sequences (11 tags in Table S5). Among the 312 sex-chromosome-specific tags, 30 unique tags were mapped to both the X and Y^h^ chromosomes (Table S3). Other tags were X (252 tags) or Y^h^ chromosome-specific (30 tags) (Table S3, Table S6). To evaluate the transcriptional activity of the genes on the X chromosomes in each flower sample, the ratio of the tag counts was calculated using the total number of X chromosome-specific tags divided by the total number of tags analyzed (Table S7). This ratio ranged from 0.47 to 0.61% (Table S7), showing no significant differences in the transcriptional activity of the X-chromosome-specific genes among the sex types.

**Figure 2:**
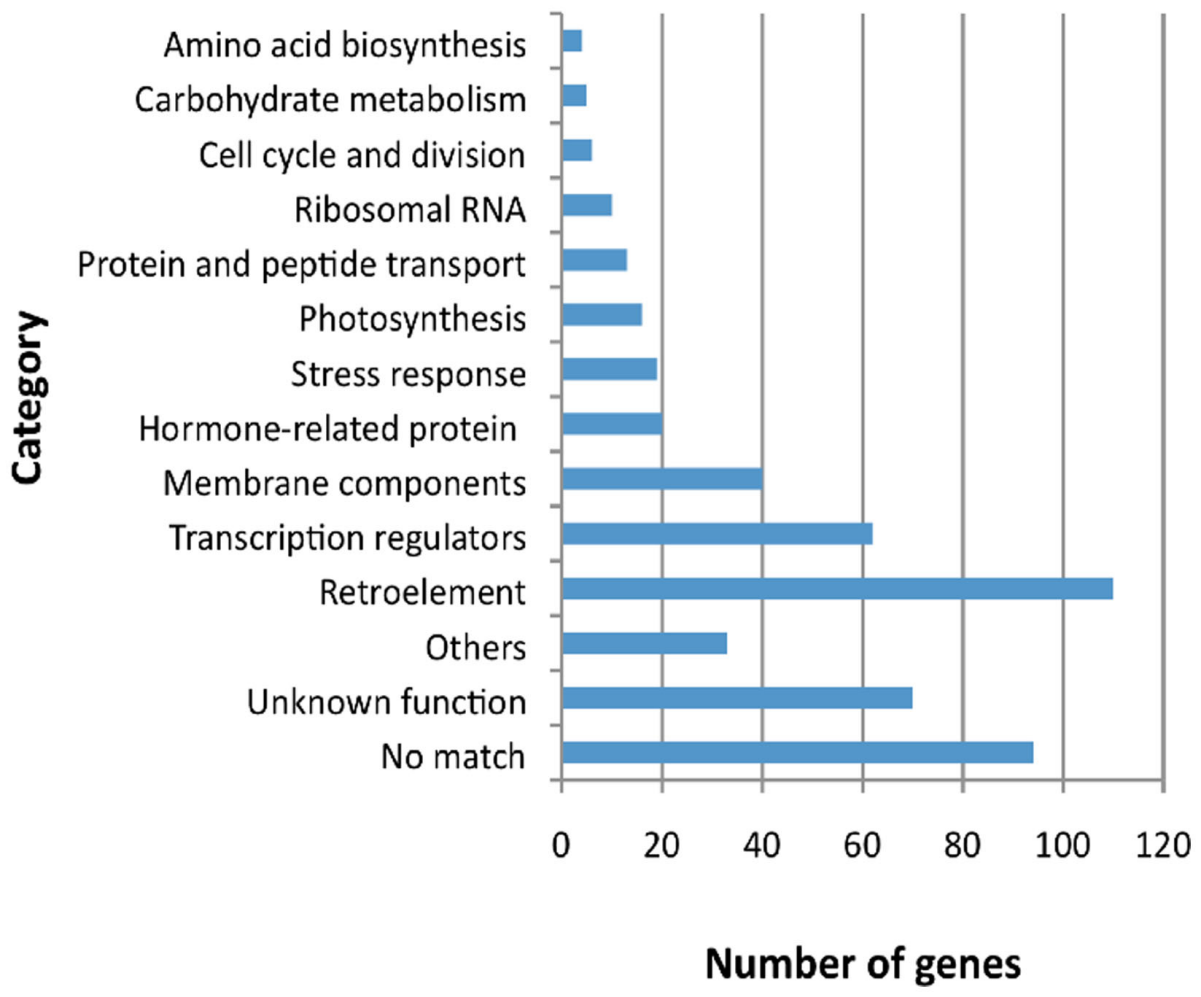
Functional categories of the genes annotated from the SC-tags. Genes corresponding to 456 SC-tags were categorized according to their function, based on their annotation as described in the Methods section. “No match” indicates the tags that had no significant similarities to any non-redundant protein sequences in the databases. “Unknown function” indicates those genes for which the functions of their encoded proteins could not be determined. The SC-tags with different annotations were grouped into the corresponding categories.

To identify their corresponding genes, these SC-tags were subjected to BLAST searches against the sequences of predicted genes from the draft genome sequence [12] or EST database of papaya. Among the 312 tags, 71 and 94 tags were perfectly matched to the predicted genes and EST sequences, respectively (Table S3). The SuperSAGE tags were likely to be located in 3′-untranslated regions [20], which might not be involved in the sequences of the predicted genes or ESTs. Therefore, we tried to find the predicted gene located near the SC-tag, revealing that 138 predicted genes were mapped within a 2 kb region upstream of the tags (Table S3). Nevertheless, the genes corresponding to many of the SC-tags remained undefined. Then, we employed a different protocol [24] to identify the genes that corresponded to the SC-tags. Approximately 1 or 2 kb of the genomic sequence upstream of the tags was subjected to a BLASTX search against the registered protein sequences in GenBank. Consequently, most of the genes corresponding to the SC-tags were annotated using this protocol, with the exception of 30 tags. These annotated SC-tags were categorized based on the predicted function of the corresponding genes (encoding proteins) (Figure 2). More than 20% of the SC-tags (110 tags) were derived from retroelements. According to their mapped positions, the retroelements were located throughout the sex chromosomes and were frequently observed in the sequences of other chromosomes (Table S4). The second most abundant group of SC-tags was the transcriptional factor group, which included the MADS-box and zinc-finger proteins. Although the SC-tags also corresponded to various other gene categories, many of the genes were functionally unknown or did not show significant similarity to any of the annotated genes in GenBank at the amino acid level.

### Differential Expression of Sex Chromosome Genes Among Sex Types

From the 312 sex-chromosome-specific SC-tags, we selected 47 tags (Table S8) that were specifically expressed in either one or two sex types. These tags were designated as sex-dependent SC-tags. Most of the sex-dependent SC-tags showed expression in both males and hermaphrodites (tags mapped to Y^h^) or in both females and hermaphrodites. Only a few tags were expressed uniquely in males or hermaphrodites, but no female-specific expressed tags were observed in this analysis.

Within the sex-dependent SC-tags uniquely mapped to the Y^h^ chromosome (15 tags), 12 tags showed expression in the male flowers carrying the Y chromosome, which was potentially due to the sequence similarity between the Y and Y^h^ chromosomes. Approximately 1 kb of the genomic sequences upstream of these Y^h^ -specific tags, except for the retroelement tags (Cp7929, Cp15892 and Cp21244), was subjected to a BLASTN search against the BAC clone sequences of the X chromosome (Table S2) to identify the alleles of their corresponding genes. From this analysis, we identified genes with significantly similar (>80% similarity) sequences to the genes for Cp12204 and Cp14501 on the X chromosome. The remaining genes were regarded as Y^h^ (Y) chromosome-specific loci, as determined using *in silico* analysis.

**Figure 3:**
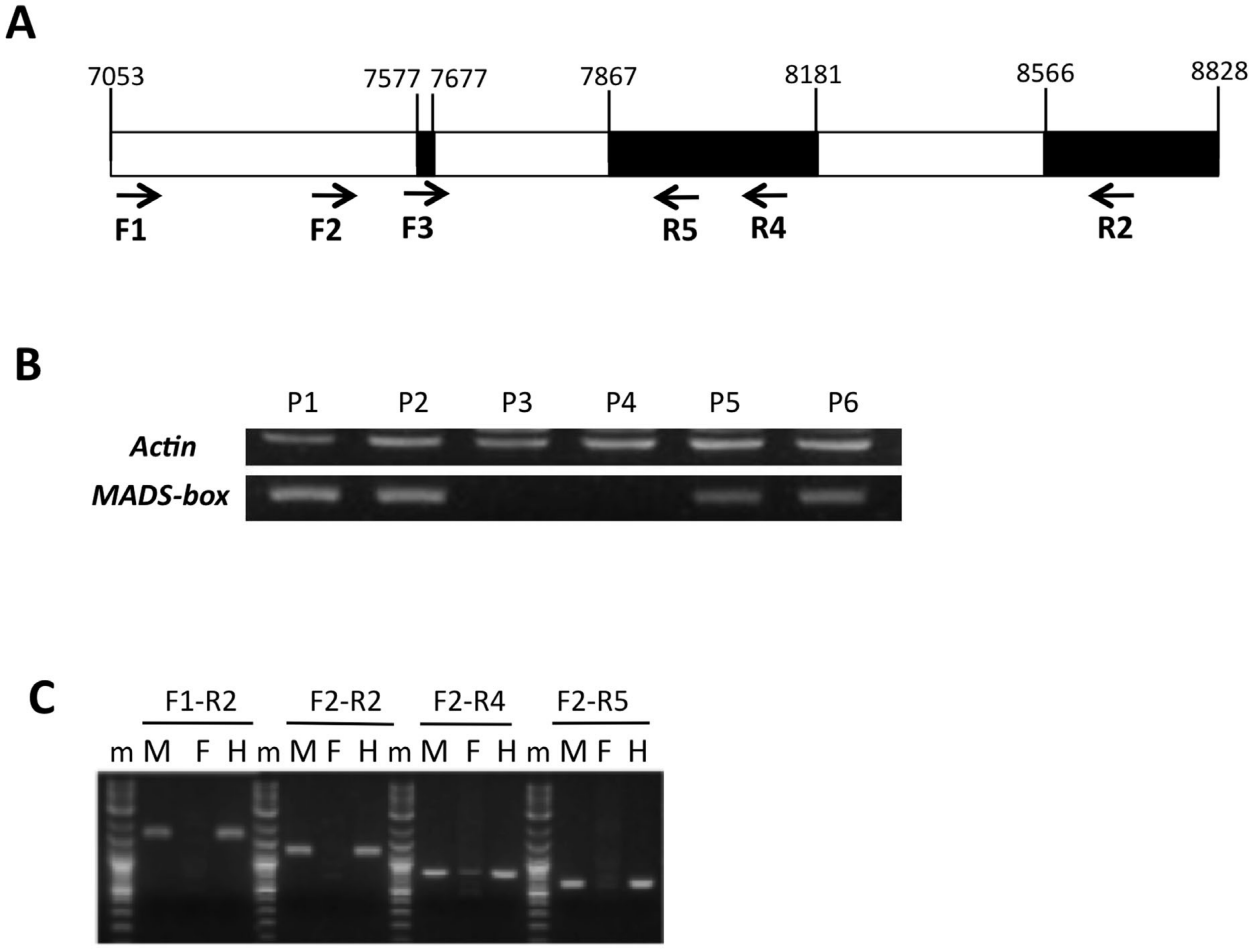
Confirmation of sex-dependent gene expression for Cp2671, which encodes a putative MADS-box protein.

We attempted PCR analysis of the genes for the two Y^h^-specific tags, Cp2671 and Cp12204, to validate their presence or absence on the X chromosome. The annotation analysis revealed that the gene corresponding to Cp2671 encoded the MADS-box protein and the gene corresponding to Cp12204 encoded monodehydroascorbate reductase (MDAR). RT-PCR analysis of the Cp2671 gene demonstrated its specific expression in male and hermaphrodite flower samples (Figure 3B, Figure S2), as shown in the data obtained from the SuperSAGE analysis. Genomic PCR amplification using several different primer sets confirmed the absence of this gene from the female genome (Figure 3A and 3C). These results demonstrated that the MADS-box protein gene corresponding to Cp2671 was uniquely present on the Y^h^ and Y chromosomes but not on the X chromosome.

**Figure 4:**
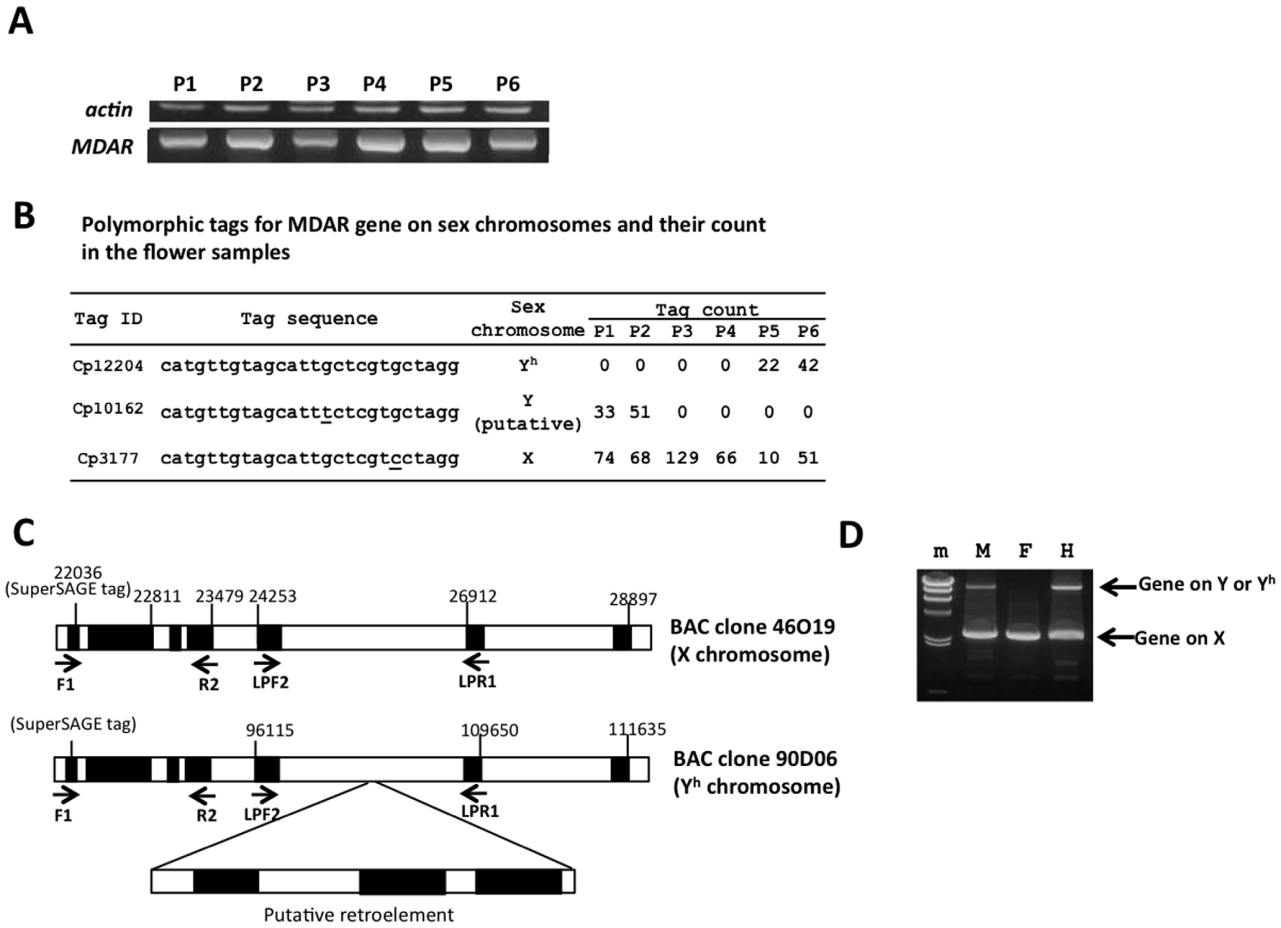
Structure and expression analysis of the genes corresponding to the Cp3177 tag, which encodes a putative monodehydroascorbate reductase (MDAR). A) RT-PCR analysis of the MDAR gene corresponding to the Cp3177 tag. P1 to P6 correspond to the flower samples indicated in Figure 1. An actin gene was used as a constitutively expressed control gene [32]. B) A list of the polymorphic tags and their counts for the MDAR genes in the Ht-SuperSAGE data. The polymorphic sequences among the tags are underlined. C) The regions flanking the Cp3177 tag on the BAC clones 46O19 (X chromosome) and 90D06 (Y^h^ chromosome). The arrows indicate the locations of the PCR primers used for amplification. The black regions represent the predicted exons. An insertion of the retroelement sequence was observed in the 90D06 sequence (Y^h^ chromosome). D) Genomic PCR amplification of the MDAR gene in each sex type. The smaller band was equally amplified in all of the sex types. The larger bands, indicating the insertion of retroelements, were only observed in males and hermaphrodites.

Although the Cp12204 tag showed hermaphrodite-specific expression, the cDNA of its corresponding gene, encoding MDAR, could also be amplified from both the male and female samples (Figure 4A, Figure S2). Amplification of the genomic sequences of the MDAR gene revealed polymorphisms among the three sex types within the SuperSAGE tag region (Figure 4B). In the current data obtained from the SuperSAGE analysis, the polymorphic tags were identified as Cp3177 and Cp10162, which were most likely derived from the alleles of this gene on the X and Y chromosomes, respectively. The Cp3177 tag was uniformly expressed in all of the samples, while Cp10162 showed male-specific expression (Figure 4B). Consequently, this MDAR gene was present on all of the sex chromosomes, and there were sequence polymorphisms present in the tag sequences among its alleles. In addition, a retroelement insertion was observed in the MDAR gene based on the BAC clone (90D06) sequences of the Y^h^ chromosome (Figure 4C). However, based on the BAC clone (46O19) sequence, there was no insertion in the corresponding allele (Cp3177) on the X chromosome (Figure 4C). PCR analysis of this gene showed that bands of approximately 2 kbp in size were amplified from all of the sex types and that an additional larger fragment was also amplified in the males and hermaphrodites (Figure 4D). This result suggests that an intact allele of the MDAR gene is present on the X chromosome and that the Y and Y^h^ chromosomes carry the allele containing the retroelement insertion.

Several tags specifically mapped to the X chromosome also showed sex-dependent expression, even though all of the sex types should carry an X chromosome (Table S8). Among these tags, the gene corresponding to the most abundant Cp11249 tag (which encodes a putative zinc-finger protein) was further analyzed. Using RT-PCR and sequence analysis of the fragments amplified from the genomic DNA, we showed that the differential expression observed in the SuperSAGE analysis might reflect sequence polymorphisms between the males and the other sex types (Figure S1). This result indicated that there are sequence polymorphisms in the X chromosome between the cultivars used in the present study. Nevertheless, several X chromosome-specific tags showed differential expression between females and hermaphrodites, which should carry the same X chromosome, indicating that the genes for these tags might be regulated at the transcriptional level.

## Discussion

We employed the previously established Ht-SuperSAGE analysis [20] in combination with SOLiD sequencing technology as tools for the transcriptome analysis of papaya flowers. We succeeded in narrowing the target genes for further analysis through the allocation of thousands of tags to either the X or Y^h^ chromosome. Thus, the SuperSAGE analysis could identify the genes expressed in the MSY regions on the X and Y^h^ chromosomes, which were difficult to predict using the genomic sequences. Consequently, we were able to select 47 differentially expressed genes that map to the sex chromosomes (sex-dependent SC-tags), which were good candidate genes for elucidating the mechanism of sex determination in papaya.

According to the model of plant sex-chromosome evolution [14], [15], the X and Y chromosomes diverged from the same origin, but many genes on the Y chromosome were lost or inactivated due to deleterious mutations. In the papaya genome, the MSY region of the Y^h^ chromosome was previously shown to have significant sequence similarity to the Y chromosome [11], which is degenerated and forms heterochromatin structures [25] that repress transcription in this region. In the draft genomic sequence of transgenic papaya, 254 genes were putatively located in the X-specific region [12], and 26 potential genes were putatively located in the MSY region of five BAC clone sequences (approximately 700 kb) [10]. The results of the SuperSAGE analysis also showed fewer Y^h^ chromosome-specific tags (30 tags) than X chromosome-specific tags (252 tags); a limited number of tags (30 tags) were commonly mapped to both the X and Y^h^ chromosomes. Although directly associating the SC-tags with predicted genes on the sex chromosomes remained difficult, the gene prediction and transcriptome data indicated the loss of functional genes on the Y^h^ chromosome.

Regardless of the degeneration and gene loss, the Y (Y^h^) chromosome is predicted to possess a dominant mutant allele for the female-sterility gene and a wild-type allele for the male-sterility gene [14]. We identified several putative genes for Y^h^-specific tags that were absent from the X chromosome. These genes might involve the wild-type allele for the male-sterility (female determination) locus because this allele was absent from the X chromosome, leading to male sterility in the absence of the Y or Y^h^ chromosome. Indeed, the experimental results definitively demonstrated that the MADS-box gene, which corresponded to the Cp2671 tag, was Y and Y^h^ chromosome-specific. In *Silene latifolia*, the presence of the Y chromosome determines sex through the regulation of floral organ development [26]. A putative stamen-promoting factor (SPF) gene on the Y chromosome has been suggested to repress the expression of the Superman-like gene (*SISUP*), which is responsible for female organ development [27] and is also a suppressor of the gene for stamen elongation (*SLM2*). The MADS-box gene, which corresponds to Cp2671, encodes a protein with 85% similarity to the Short Vegetative Phase (SVP) protein in *Arabidopsis* [28] (data not shown), which is a well-known transcriptional regulator of the gene for flowering time (*FT*) [29]. Although this MADS-box protein potentially plays a role in the trans-regulation of other genes, further analysis, including gene knockdown in the male or overexpression in the female, is required to determine its role in papaya sex determination.

A dominant mutation of the female-sterility gene on the Y chromosome is predicted to control male sex determination [14]. Thus, an allele of this locus should also be present on the X chromosome. Allele dominance is rendered through the predominant expression of the allele or the increased activity of its gene product. The results from the transcriptome analysis revealed that the gene corresponding to the Cp14501 tag was specifically expressed in male and hermaphrodite flowers, but based on the BLAST analysis, its allele was potentially present on the X chromosome, suggesting that this gene is a candidate female-sterility gene. However, we also have to consider the differences in the sequences of the genes expressed among the alleles on the sex chromosomes as causal mutations of female sterility (male determination).

During the early stages of development, the male flower was quite similar to that of the hermaphrodite (Figure 1); thus, the female-sterility (male determination) allele on the Y chromosome would suppress the maturation of female organs at a later stage. In hermaphrodites carrying the Y^h^ chromosome, an additional mutation at an independent or female-sterility locus possibly represses the function of the female-sterility allele to promote female organ development. To identify the male- and hermaphrodite-determination genes described above, the genomic sequence of the MSY on the Y chromosome should be deciphered and compared with a similar region on the X or Y^h^ chromosome.

The retroelement was the most frequently observed gene group among the transcripts from the sex-chromosome regions in the papaya flowers. Zhang *et al.* [25] observed four knob-like heterochromatin structures that were specific to the MSY and revealed that the DNA sequences associated with the heterochromatic knobs were heavily methylated compared with the sequences in the corresponding X-chromosome domains. The retroelements were assumed to undergo heterochromatinization. However, the results presented here demonstrate that some of these retroelement genes, specifically those located on the sex chromosomes, are at least transcriptionally active. Among these retroelement tags, the Y^h^-specific Cp15892 tag was also expressed in the female samples, implying a potential transposition from Y^h^ to any other chromosome. The putative inactivation of genes by retroelement insertion was observed in the MDAR gene on the Y and Y^h^ chromosomes, which likely promotes sequence divergence between its alleles among the sex chromosomes. MDAR has been suggested to be crucial for cell viability because of its reactive oxygen scavenging activity [30]; therefore, the lack of an intact MDAR allele on the X chromosome might result in lethality.

In X/Y chromosome sex determination, the gene dosage on the X chromosome is doubled in females (XX), and the expression of these genes is compensated through the inactivation of one of the X chromosomes in female mammals [31]. In papaya, a dosage effect (increased expression in females) was not observed for several X-linked genes [11]. The results from the SuperSAGE analysis show a limited number of tags on the X chromosome with sex-dependent expression. However, 87% of the tags on the X chromosome (220/252) were expressed in all of the sex types, and the transcriptional activity from the genes on the X chromosome was equal among all of the samples. This result implies dosage compensation of the genes on the papaya X chromosome. The gene expression analysis of the different flower samples showed that gene transcription was strongly regulated via developmental signals in the flower organs. Therefore, a comparison of the transcriptomes of similar tissues, such as the leaves, should be performed among the sex types during development. In addition, the polymorphisms in the genes expressed on the X chromosome between cultivars will be useful indicators for evaluating the transcript levels in the female.

In the present study, we analyzed the transcriptome of genes on the papaya sex chromosomes and identified the MADS-box gene for Cp2671 as a candidate for sex determination because of its presence only on the Y and Y^h^ chromosomes. To determine its role in sex determination, further functional analysis of this gene, including genetic transformation in papaya, is necessary. Furthermore, the other genes for sex determination might be present in the results obtained from the transcriptome analysis performed in this study; therefore, the structural and functional analysis of those genes will be required to better understand sex determination in papaya.

## Materials and Methods

### Plant Materials and RNA Preparation

The plant materials used in this study were obtained from the Hawaiian papaya cultivar “Sunrise solo”, and the breeding material TM1 was derived from a cross between cv. “Wonder frea” and Okinawan land race IG4. The female and hermaphrodite flower buds were harvested from the Sunrise solo cultivar, and the male flower buds were obtained from TM1. The flowers in the early (no longer than 7 mm) and late (approximately 20 mm in length) developmental stages were harvested from one-year-old plants of the three sex types grown in a greenhouse. To avoid a high-temperature-induced sex change, the samples were collected in March and April (spring season). Each developmental stage of the flower sample from each sex type was designated as P1 to P6 (Figure 1). Twenty early-stage flowers (P1, P3, P5) or three late-stage flowers (P2, P4, P6) from each sex type were pooled and subjected to total RNA extraction using the RNeasy Plant Mini Kit (Qiagen). For the cDNA synthesis of SuperSAGE, 10 µg of total RNA per sample were used.

### Adapter Preparation

For the adapter-2-SLD, two oligonucleotides (5′-TTCCTCATTCTCTCAAGCAGAAGACGGCATACGAAATGATACGGCGACCACCGACAGGTCTAACGATGTACGCAGCAGCATG-3′ and 5′-CTGCTGCGTACATCGTTAGACCTGTCGGTGGTCGCCGTATCATTTCGTATGCCGTCTTCTGCTTGAGAGAATGAGGAA-amino-3′) were synthesized and annealed. For the adapter-1-SLD, two oligonucleotides (5′-CCACTACGCCTCCGCTTTCCTCTCTATGGGCAGTCGGTGATXXXXXX-3′ and 5′-NNXXXXXXATCGTCGGACTGTAGAACTCTGAACCTGT-amino-3′; XXXXXX denotes variable index sequences, as shown in Table 1) were synthesized and annealed.

### Tag Extraction and Preparation of Sequencing Templates

Double-stranded cDNA was synthesized using the biotinylated adapter-oligo(dT) primer (5′-bio-CTGATCTAGAGGTACCGGATCCCAGCAGTTTTTTTTTTTTTTTTT-3′). Purified cDNA was digested with NlaIII. The resulting fragments were bound to streptavidin-coated beads (Dynabeads streptavidin M-270), and the non-biotinylated cDNA fragments were removed by washing. Adapter-2SLD was ligated to the cDNA fragments on the beads and digested with EcoP15I after washing. EcoP15I-digested and released fragments (adapter-2SLD-tags) were ligated to adapter-1SLD with defined index sequences for sample identification.

The tags located between the two adapters were amplified using PCR with PhusionHigh polymerase and the P1 (5′- CCACTACGCCTCCGCTTTCCTCTC -3′) and P2 (5′- CTGCCCCGGGTTCCTCATTCTCTCAAGCAGAAGA -3′) primers. The PCR program consisted of denaturation at 98°C for 1 min followed by 12 cycles of 98°C for 30 sec and 60°C for 30 sec. Eight tubes from this PCR amplification (15 µl each) were pooled, and the concentrated PCR products were purified using the MinElute reaction purification kit (Qiagen) and separated on an 8% non-denaturing polyacrylamide gel. After staining with SYBR green (Takara Bio), the 160-bp band was excised from the gel, and the DNA was eluted from the gel pieces. The PCR product from each sample was analyzed on an Agilent Bioanalyzer 2100. Equal quantities of the PCR products from all samples were mixed, and the mixture was analyzed using the Applied Biosystems SOLiD3.

### Sequencing

The purified and mixed PCR products were applied to an emulsion PCR. The extraction and purification of the templated beads from the emulsion PCR mixture were performed according to the manufacturer’s instructions. A total of 42.5 million templated beads were applied to a well in the quad deposition chamber of the SOLiD3 system. The sequencing was performed according to the protocol in the instrument operation guide.

### Data Analysis

The CSFASTA-formatted sequence files from SOLiD3 were converted toFASTA-formatted files for further analysis. The sorting of the sequence reads was based on the indexed sequences, and the subsequent extraction of the sequence tags from the reads was conducted using a script written in Perl [20]. The tag profiling data (list of tag sequences and their counts) were registered in the NCBI Gene Expression Omnibus under the accession number GSE30366.

Sequences of *Carica papaya* whole genome, predicted genes and ESTs were downloaded from the FTP site (ftp://ftp.plantgdb.org/download/Genomes/CpGDB/). The BAC clone sequences of the sex chromosomes (Table S2) were also downloaded from the NCBI website from previous studies of papaya [8], [10], [11]. The tag sequences that appeared more than 10 times in all the samples were used as queries in BLASTN searches against the BAC clone sequences for the sex chromosomes or papaya genomic sequences (nuclear and organelle genomic sequences) to investigate redundancy. Only the tags showing a perfect match with the sequences for the sex chromosomes were regarded as SC-tags (sex-chromosome tags). Although tags showing a single base-pair mismatch were also identified, we did not include them in the present analysis. The SC-tags were also mapped to draft genomic sequences (supercontigs) and predicted genes were found within 2kb upstream of the SC-tags according to their location in the draft genome. For the annotation of the genes corresponding to the SC-tags, 2 kb of the genome sequence upstream of the 5′-CATG in each individual tag sequence was used as a query for a BLASTX search against the registered protein sequences in GenBank. When no significantly similar proteins were identified, an additional 1 kb of the genomic sequence upstream of the tag sequences was used as the BLASTX query.

### PCR Amplification of Genes Corresponding to the Tags

Genomic DNA was extracted from the mature leaf of each papaya plant using the DNeasy Plant Mini Kit (Qiagen). For the RT-PCR analysis, two pools from each flower sample (P1–P6) were harvested from independent plants and subjected to total RNA extraction. Single-stranded cDNA was synthesized from the total RNA by reverse transcription with the adapter-dT primer (5′-GCTGTCAACGATACGCTACGTAACGGCATGACAGTG(T)_24_-3′, Invitrogen). These cDNAs were used as templates for the RT-PCR analysis (Figure S2).

The primer sequences for the amplification of the genes corresponding to the Cp2671 tag (MADS-box protein) are listed as follows: Cp2671-F1 (5′-GAGATGCACCATTCTAGTTCC-3′), Cp2671-F2 (5′-CAGATCGGGCACTTTAATGTTG-3′), Cp2671-F3 (5′-TGGCAAACTCTGTACTTGCA-3′), Cp2671-F3 (5′-TGGCAAACTCTGTACTTGCA-3), Cp2671-R2 (5′-GGAGCTCCAAGGATTAAGCA-3′), Cp2671-R4 (5′-GCAGGGAATACAATTGATGG-3′) and Cp2671-R5 (5′-TCGGAGAATGTAGCGTCTGA -3′). For the amplification of genes corresponding to Cp3177 (MDAR gene), the primers Cp3177-LPF1 (5′-GTGTGAAATGATGGAAGCCGTGCACGAGCTGCAGATGAAT-3′), Cp3177-LPF2 (5′-CGTGTTCCAAGAACTAACTCAATTCCTAAGAAAATATGCGGAG-3′), Cp3177-F1 (5′-GAGTGATGATGTAACAAGTTAG-3′) and Cp3177-R2 (5′-CATGGTGTTCCCTGAAGCAC-3′) were employed. As a control, the amplification of an actin gene was performed using the primers Cp47F (5′-GTCCTAGACACACAGGTGTCATGG-3′) and Cp47R (5′-GACTTGTCCATCAGGCAACTCGTA-3′). For the RT-PCR shown in Figure S1, the primer sequences used are described in each file.

## Supporting Information

Figure S1 **RT-PCR analysis of the gene for Cp11249.** A) Partial fragment of the gene for Cp11249 (encoding putative zinc finger protein) was amplified from male (P1), female (P3) and hermaphrodite (P5) cDNA. In the lane “m”, a 100 bp ladder size marker was loaded. Template cDNA was prepared as described in the Method. The Cp11249F (5′- CATATATGGATTGGGGAAAC-3′) and Cp11249RV (5′- ACCTGGAGCTGTATGGTAAGATT -3′) primers were used for amplification. B) RT-PCR experiments with primers for the partial Cp11249 gene in two additional replicated flower samples (P1, P3 and P5). In the lane “m”, a lambda HindIII digested size marker was loaded. C) An amplified fragment from each sample (P1, P3 and P5) was applied to the sequencing analysis, and aligned. The Cp11249 tag sequence is shown above the alignments, and the tag regions in the cDNA sequences are surrounded by a square. The SNP in P1 (male) was underlined.
(PPT)

Figure S2 **RT-PCR analysis of three genes in replicated flower RNA samples.** RT-PCR experiments with primers for the MADS-box protein gene (for Cp26719) as shown in Figure 3 and for the MDAR gene (for Cp12204) as shown in Figure 4 were repeated in two independently replicated RNA samples from each papaya flower sample (P1 to P6).
(PPT)

Table S1 List of analyzed SuperSAGE tags from papaya flowers*. * Count for individual tag from all six samples (P1 to P6) were combined and indicated as “SUM” in the table, and only the tags, showing more than 10 counts of “SUM”, were listed in this table.
(XLS)

Table S2 **BAC clones of the papaya sex chromosomes used for mapping of SuperSAGE tags.** * These clones were regarded as BAC clones on the X chromosome.
(XLS)

Table S3 **SuperSAGE tags specifically mapped on the sex chromosome (SC-tags).** * Predicted gene or EST, which showed perfect match to 26-bp tag sequence. ** Predicted gene, which was found within 2 kbp upstream of the tag sequence. *** Deduced proteins were determined by BLASTX searching of genomic sequences upstream of the SuperSAGE tags as described in method.
(XLS)

Table S4 **SuperSAGE tags mapped on the sex chromosome and other chromosomes simultaneously.** * Deduced proteins were determined by BLASTX searching of genomic sequences upstream of the SuperSAGE tags as described in method.
(XLS)

Table S5 **SuperSAGE tags mapped on the sex chromosome and organelle genome sequences simultaneously.** * Deduced proteins were determined by BLASTX searching of genomic sequences upstream of the SuperSAGE tags as described in method.
(XLS)

Table S6 **Expression patterns of SC-tags among male, female and hermaphrodite.** *Sex types are designated as M for male, F for female and H for hermaphrodite. **SC-tags were mapped on both X and Y^h^ chromosome.
(XLS)

Table S7 **Transcription activity of genes mapped on the X chromosome among papaya flowers from different sex types.**
(XLS)

Table S8 **List of the sex dependent SC-tags.** * Deduced proteins were determined by BLASTX searching of genomic sequences upstream of the SuperSAGE tags as described in method.
(XLSX)
