# Supplementary material for: Digital Transcriptome Analysis of Putative Sex-Determination Genes in Papaya (Carica papaya)
Source: PLoS One. 2012 Jul 16;7(7):e40904. doi: 10.1371/journal.pone.0040904 (PMC3397944; doi:10.1371/journal.pone.0040904)
Supplement: Figure S1 — RT-PCR analysis of the gene for Cp11249. A) Partial fragment of the gene for Cp11249 (encoding putative zinc finger protein) was amplified from male (P1), female (P3) and hermaphrodite (P5) cDNA. In the lane “m”, a 100 bp ladder size marker was loaded. Template cDNA was prepared as described in the Method. The Cp11249F (5′- CATATATGGATTGGGGAAAC-3′) and Cp11249RV (5′- ACCTGGAGCTGTATGGTAAGATT -3′) primers were used for amplification. B) RT-PCR experiments with primers for the partial Cp11249 gene in two additional replicated flower samples (P1, P3 and P5). In the lane “m”, a lambda HindIII digested size marker was loaded. C) An amplified fragment from each sample (P1, P3 and P5) was applied to the sequencing analysis, and aligned. The Cp11249 tag sequence is shown above the alignments, and the tag regions in the cDNA sequences are surrounded by a square. The SNP in P1 (male) was underlined. (PPT) [file pone.0040904.s001.ppt]

## Slide 1
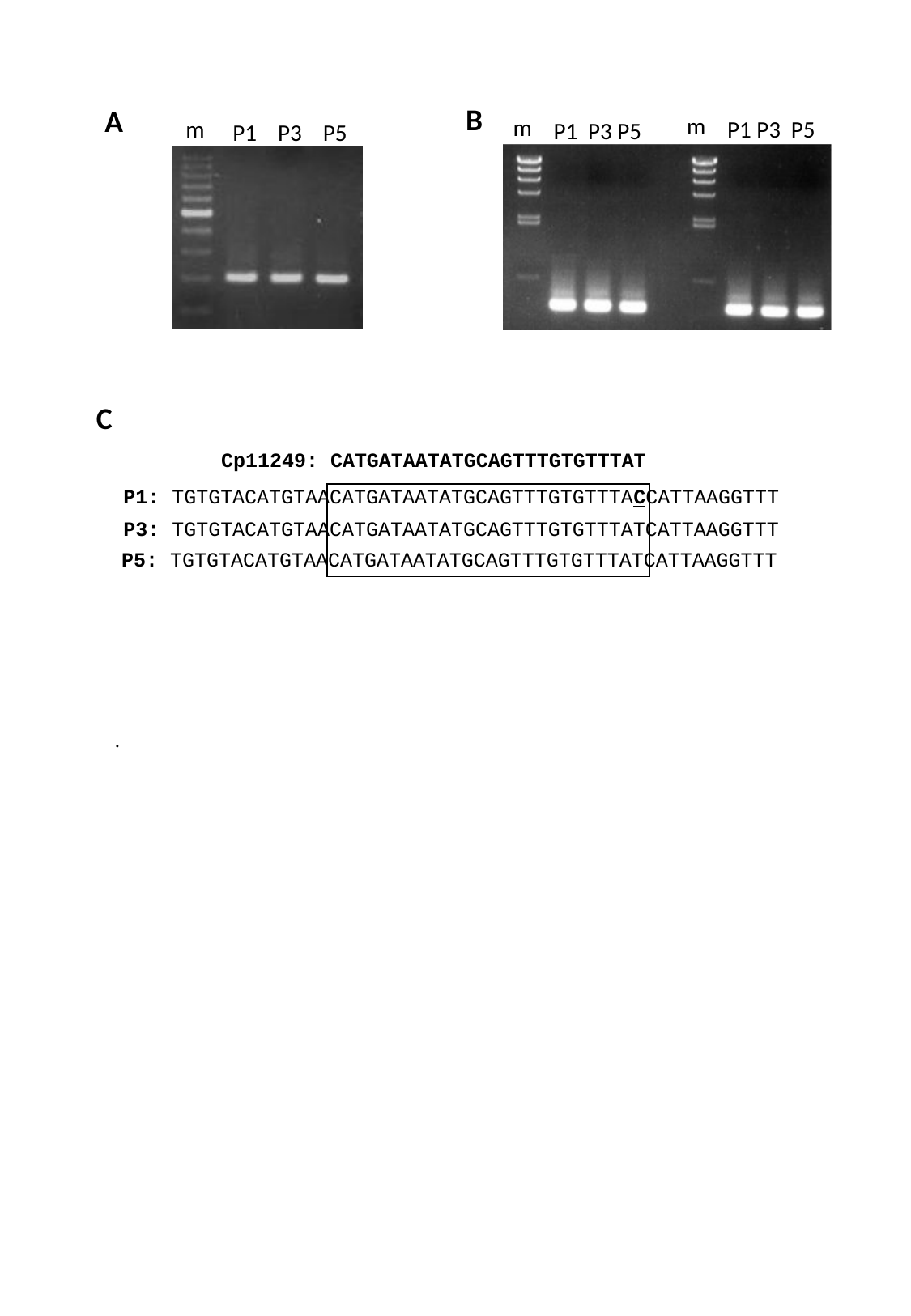

B
A
m
m
m
P1 P3 P5
P1 P3 P5
P1 P3 P5
C
Cp11249: CATGATAATATGCAGTTTGTGTTTAT
P1: TGTGTACATGTAACATGATAATATGCAGTTTGTGTTTACCATTAAGGTTT
P3: TGTGTACATGTAACATGATAATATGCAGTTTGTGTTTATCATTAAGGTTT
P5: TGTGTACATGTAACATGATAATATGCAGTTTGTGTTTATCATTAAGGTTT
.
