# Supplementary figures and images for: Digital Transcriptome Analysis of Putative Sex-Determination Genes in Papaya (Carica papaya)
Source: PLoS One. 2012 Jul 16;7(7):e40904. doi: 10.1371/journal.pone.0040904 (PMC3397944; doi:10.1371/journal.pone.0040904)

## Slide 1
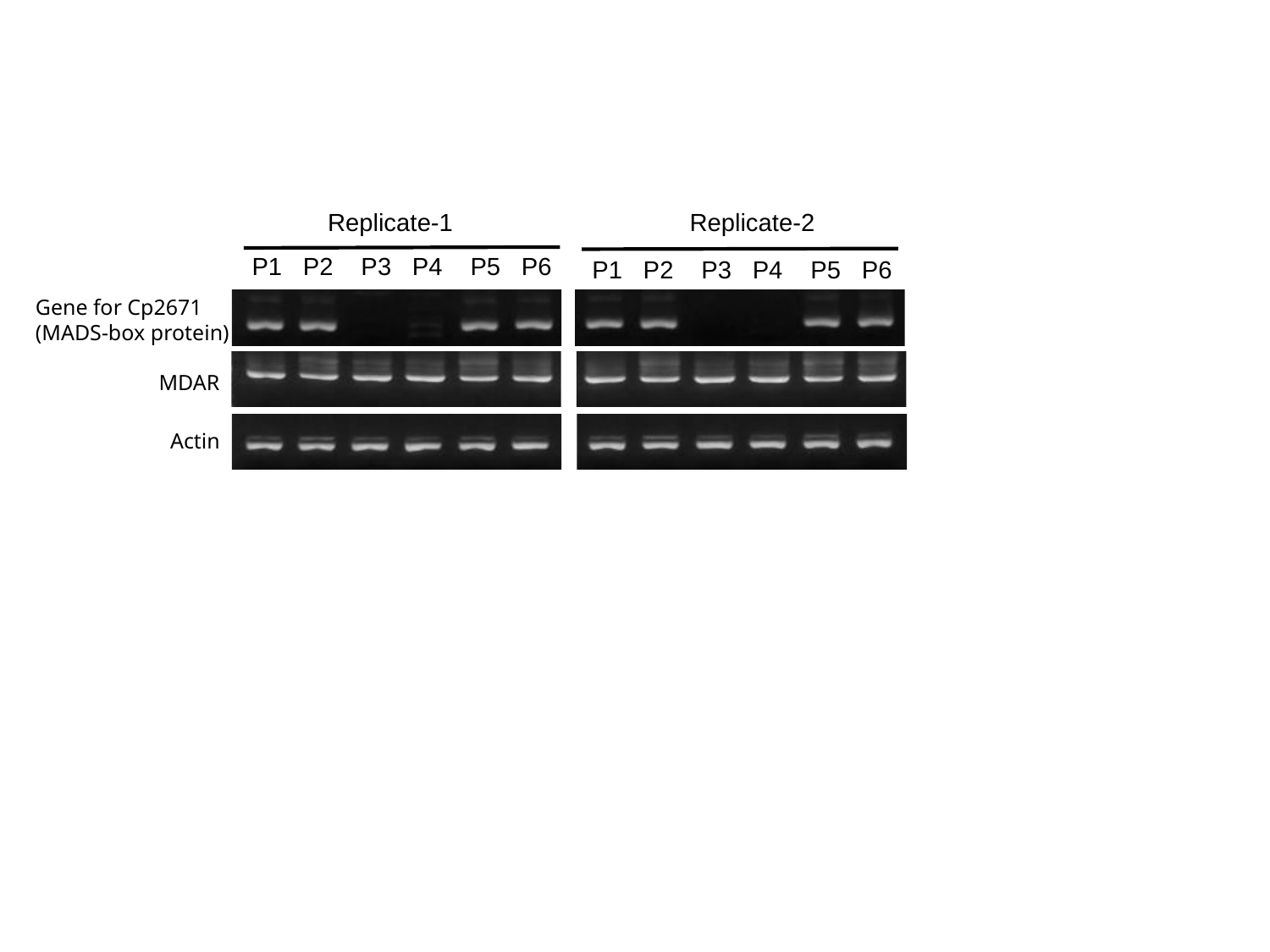

Replicate-1
Replicate-2
P1 P2 P3 P4 P5 P6
P1 P2 P3 P4 P5 P6
Gene for Cp2671
(MADS-box protein)
MDAR
Actin

Supplement: Figure S2 — RT-PCR analysis of three genes in replicated flower RNA samples. RT-PCR experiments with primers for the MADS-box protein gene (for Cp26719) as shown in Figure 3 and for the MDAR gene (for Cp12204) as shown in Figure 4 were repeated in two independently replicated RNA samples from each papaya flower sample (P1 to P6). (PPT) [file pone.0040904.s002.ppt]
